# New era for *Tropical Medicine and Health*

**DOI:** 10.1186/s41182-016-0001-1

**Published:** 2016-03-14

**Authors:** Masahiro Hashizume

**Affiliations:** Tropical Medicine and Health, Institute of Tropical Medicine, Nagasaki University, 1-12-4 Sakamoto, Nagasaki, 852-8523 Japan

It is an honor and pleasure to be able to extend greetings as the Editor-in-Chief of *Tropical Medicine and Health* (*TMH*), a journal with a 56-year history of high-quality research and one of the most important publications in the field. When I was appointed to the position of Editor-in-Chief in 2014, I wrote in the editorial that “our greatest challenge is to continue to improve the journal in such a way as to provide essential and timely information regarding research in the field of tropical medicine and global health” [1]. To achieve this goal, we set up a target to shorten the submission/review/decision cycle to 21 days after the initial submission. I am delighted to report that this target was achieved in two consecutive years in 2014 and 2015: the average duration was 21.0 and 19.0 days, respectively. This achievement owes greatly to the efforts of our reviewers and editors. We believe that speedy publication will help to ensure that *TMH* papers are read and cited more often by experts in the related fields.

*TMH* is attracting ever greater attention. The number of accesses and downloads of the papers on the journal website increased 3.9 and 2.6 times, respectively, in 2015, compared to the average in 2013. We also received 46 % more submissions to *TMH* in 2015 compared to 2013. While this is due in part to our promotion campaign as well as our recent decision to send e-mail announcements to society members right after a paper is published online, we are deeply indebted to all of our authors, reviewers, and readers for their enthusiastic cooperation.

Now I am pleased to announce that BioMed Central is the new publisher of *TMH*. We have taken this opportunity to revise the aims and scope of the journal to incorporate, in addition to traditional tropical medicine, the issue of global health. Global health is health of populations in a global context which can be characterized by a “health issue that concerns many countries or is affected by transnational determinants” [2]. While traditional tropical medicine is still central to our scope, *TMH* welcomes clinical, epidemiological, laboratory, and policy research physically implemented in non-tropics when related to health issues in the tropics or in a global context.

*TMH* will continue as an open-access publication as part of BioMed Central’s high-quality portfolio of open-access journals. Authors can expect accepted manuscripts to be published online in professionally copy-edited and typeset form within a few weeks of acceptance.

Finally, I would like to convey my cordial request to all our contributors for their continued support of *TMH*. The *TMH* staff will continue to make their utmost efforts to achieve speedy publication and to provide essential and timely information on high-quality research in the field of tropical medicine and global health. On behalf of the Editorial Board and *TMH* staff, I look forward to working with you and serving the community.
